# *“It was really helpful for me, and at the same time it was really tough”*: a qualitative study among Afghan peer refugee helpers in Greece

**DOI:** 10.1186/s12888-024-06255-4

**Published:** 2024-11-16

**Authors:** Michalis Lavdas, Gro Mjeldheim Sandal, Synnøve Bendixsen

**Affiliations:** 1 Department of Psychosocial Science, Faculty of Psychology, University of Bergen, Christies gt. 12, Bergen, 5015 Norway; 2 Department of Social Anthropology, Faculty of Social Sciences, University of Bergen, Bergen, Norway

**Keywords:** Peer support, Mental health, Peer refugee helpers, Job demands, Sense of coherence

## Abstract

**Background:**

Aid workers with a refugee background are increasingly engaged in the humanitarian field. These individuals, known as peer refugee helpers (PRHs), contribute to providing psychosocial support for other refugees. However, few studies have focused on the mental health and wellbeing of PRHs.

**Objectives:**

This study aims to investigate the experiences of PRHs of Afghan origin in the humanitarian field in Greece.

**Methods:**

Seven focus group interviews took place in a hybrid format, online or in-person. PRHs of Afghan origin (*N* = 18), working for various Aid/Humanitarian Organizations (AOs), participated. Data was analyzed using template analysis.

**Results:**

The analysis identified job resources reflected in the dimensions of sense of coherence (meaningfulness, manageability, comprehensibility) and job demands associated with role overload, role conflict, and role boundaries. Gender differences were noticeable. Specifically, female PRHs experienced empowerment, through accessing new opportunities often not available within Afghan communities. Female PRHs frequently reported going against traditional gender roles and faced negative reactions from their peers. Male PRHs reported feeling empowered through their engagement as a PRH, as it helped them fulfill their personal goals, such as becoming a good example within their families or communities.

**Conclusions:**

Recommendations for policymakers and AOs based on the study results include: (1) Offer training, supervision, and psychosocial support for PRHs to enhance work engagement and prevent emotional exhaustion; (2) Address gender differences ensuring the protection and support of female PRHs; (3) Adopt scalable psychosocial interventions to promote agency and a concrete way of engaging with beneficiaries; (4) Formalize job roles outlining responsibilities and expectations for PRHs.

**Supplementary Information:**

The online version contains supplementary material available at 10.1186/s12888-024-06255-4.

## Introduction

More than 630,000 individuals are engaged as aid workers globally according to the Active Learning Network for Accountability and Performance in humanitarian action [3]. This paper focuses on a group of aid workers that has received limited attention in international research: individuals with refugee backgrounds involved in humanitarian care, referred to as Peer Refugee Helpers (PRHs) [17]. Involving refugee community members in humanitarian work is integral to delivering effective interventions [2, 25]. While it has been often assumed that engaging refugees benefits their wellbeing and self-worth [48], scientific evidence on the impact of being a PRH remains sparse. Our study aims to address this gap in the literature by examining how Afghan refugees experience their engagement as PRHs. This group was chosen, as Afghans constitute one of the largest displaced populations worldwide, as well as in Greece, where our study took place [51].

### Peer support and scalable interventions among refugees

Peer support has been widely implemented in the field of mental health and addiction [44] and is defined as *“a system of giving and receiving help founded on key principles of respect*,* shared responsibility*,* and mutual agreement of what is helpful”* ([37], p. 6). Peer support has been found to foster hope and empowerment among individuals (beneficiaries) receiving these services [34]. Peer supporters, advocate for the needs of their beneficiaries, help their beneficiaries overcome barriers at a system level, and navigate within the healthcare system [32].

Research has suggested that scalable psychosocial interventions provided by non-specialists are effective in alleviating psychological distress among refugees and asylum seekers [50] across various mental health problems [14, 17, 40], and help address integration challenges [35]. Key aspects of these interventions are that the beneficiaries and the PRH share a background of forced migration and a common language, fostering a safe and culturally attuned space for communication [35].

### Afghan PRHs in Greece

More than 1.2 million people have crossed Greece, seeking asylum in the EU [53], many of whom stayed in the country [29]. Arrivals of forced migrants in Greece have decreased significantly since 2016. However, the number of dead or missing people has grown significantly over the last five years indicating the increasing risks of undertaking the journey to Europe. For many Afghans, Greece serves as a gateway to Europe [19]. Key reasons for their onward migration to other EU countries are prolonged conflicts [2] and poor living conditions [29]. The withdrawal of international forces and the Taliban governance since 2021 - particularly loss of women’s rights, freedom of expression, and individual liberties - seem to have caused significant mental health challenges among the Afghan population [38].

In Greece, 81 Aid-Humanitarian Organizations (AOs), providing support to refugees, operated in 2022 [24]. AOs refer to Non-Governmental Organizations (NGOs), International NGOs, or other formally established entities operating in the humanitarian field in Greece (ex. grassroot organizations). Afghan PRHs connected to these AOs have been engaged in various areas such as delivering psychosocial interventions [30], becoming teachers for their own community [42], interpreters in humanitarian organizations [52], and contributing to the foundation of refugee-led organizations [41].

### The wellbeing and mental health of PRHs

There is limited empirical evidence on how assuming a helper role impacts the wellbeing and mental health of PRHs themselves. However, scholars have suggested that engaging Afghan refugees and asylum seekers in service development may facilitate access to mental health care and strengthen their social support networks [2]. Afghans living in Greek refugee camps reported that involvement in peer support was an effective strategy for alleviating depression and coping with monotony [31]. Additionally, PRHs have reported positive experiences such as learning self-management strategies and applying them in their own life while helping others [9], and experiencing a sense of accomplishment from overcoming psychological distress triggered by listening to the traumatic narratives of their beneficiaries [45].

On the other hand, PRHs further share a common background with their beneficiaries related to ongoing struggles in daily life post-migration, often associated with psychological distress [33]. Surkan et al. [47] demonstrated that failure to maintain a professional distance to beneficiaries was linked to feelings of frustration and sadness among PRHs.

While research on the wellbeing of PRHs is limited, studies on other groups of aid workers, both international and national, may be relevant. A comprehensive study investigating risk and protective factors for mental health of aid workers, highlighted that being an international worker, working in emergency settings, with low income and job insecurity may contribute to mental health problems [55]. The risk factors may be gender specific. Female aid workers may experience more discrimination and harassment compared to their male counterparts [36]. Seeking mental health support might carry a greater stigma for men [55]. To date, however, little research exists on potential gender differences within the group of PRH.

### Theoretical framework

In this paper, we examined the experience of PRHs through the lens of salutogenesis [4] and the Job Demand - Resource Model (JD-R) [7]. Salutogenesis pertains to the factors that help individuals remain healthy when facing adversity and addresses health as a continuum [10]. Sense of Coherence (SOC) is a central concept in the salutogenic approach and involves three components: (1) perceiving life as predictable and structured (comprehensibility), (2) having the resources to meet life’s demands (manageability), (3) understanding such demands as challenges and feeling adequately motivated to engage with them (meaningfulness) [5].

The JD-R model aligns with the salutogenic approach [27], highlighting the occupational stress that arises from an imbalance between work-related demands and the resources available to an individual. Job demands are defined as *“physical*,* psychological*,* social*,* or organizational aspects of the job that require sustained physical and/or psychological effort or skills and are therefore associated with certain physiological and/or psychological costs”* ([7], p. 312). Resources foster resilient responses to these demands, enable effective functioning at work, and contribute to personal growth and protecting health [18]. While job demands can increase job strain, leading to emotional exhaustion and burnout, job resources facilitate work engagement from a motivational perspective. Job resources are assumed to moderate the effect of job demands on wellbeing by reducing emotional exhaustion [7]. Bakker and Demerouti [8] found that personal resources, including self-efficacy, optimism, and organizational-based self-esteem, act as buffers against job demands and contribute to employee resilience and engagement.

Job demands such as role overload, role conflict, and role ambiguity, may have an excessively constraining impact on individuals [8]. An extensive literature suggests that these job stressors are linked with negative health effects [6, 15, 20, 23]. Role overload pertains to the demands stemming from one or several roles for which an individual lacks the adequate resources to navigate successfully [20]. Hecht ([23], p. 112) suggested that role conflict occurs “*when demands associated with one role interfere directly with one’s ability to satisfy the demands of another role*”. For example, role conflict may pertain to incompatible expectations from work and the family. Role ambiguity, stems from unclear goals and expectations, hindering individuals from effectively fulfilling their job role [15]. These demands have previously been studies in the context of peer support work [16].

The Sense of Coherence (SOC) reflects the resources individuals possess to manage work-related stressors, moderating the effect of demands on individuals and facilitating work engagement [54]. Measures of SOC have been applied in previous research in humanitarian settings. For instance, SOC was identified as a protective factor for the mental health of Palestinian aid workers. The researchers observed that SOC enabled these workers to cope with adversity and compensated for their limited specialized education and training [56].

### Aims of the study

To our knowledge, no research has comprehensively explored what fosters and hinders PRHs’ mental health and wellbeing in the humanitarian field. In this study, we aim to explore the experiences of Afghan PRHs, formally affiliated with AOs in Greece in paid or non-paid positions, using a qualitative approach. Through this research, we seek to provide insight into the job-related demands and sense of coherence (SOC) as a resource relevant to the PRHs’ mental health and wellbeing.

## Method

### Design

This study adopts an exploratory qualitative design grounded in a critical realist epistemological position, using a theory-driven approach to deepen the understanding of the PRHs’ experiences. Our starting point is a strength-based theoretical framework that aligns with salutogenesis and explores the factors that help individuals remain healthy. We integrated theoretical concepts that would best highlight the resources and challenges of PRHs consistent with our epistemological position [21]. In this context, we explored the narratives of PRHs, acknowledging the interplay between personal, organizational, and sociocultural factors. We used semi-structured focus group interviews, where PRHs could interact with each other and explore themes through multiple perspectives that were guided by our wider initial theoretical framework. A participatory approach through a reference group and two PRHs acting as cultural mediators further informed the contextualization of the PRHs’ narratives.

### Participants

Participants (*N* = 18, 9 female participants) were providing paid or non-paid work for an AO in Greece at the time. Fourteen of the participants worked in camps, and four in urban areas. Purposive sampling was used for the recruitment. Inclusion criteria for participating in the study included being an adult of Afghan origin, having a refugee or asylum seeker status, and speaking Dari or Farsi. Additional background information about the participants can be found in Table 1. The study had a balanced number of male and female PRHs, with most participants being between 25 and 35 years old. We included both refugees and asylum seekers. The newly arrived were exclusively women (0–3 years in Greece), while the men had been in Greece between three and five years.

**Table 1:** Demographic characteristics of participants

Characteristics	Gender	
	Female (*n* = 9)	Male (*n* = 9)
Age (range)^a^		
20–25 years	1	
25–30 years	3	5
30–35 years		3
35–40 years	1	
40–45 years	2	
45–50 years		1
Country of origin		
Afghanistan	8	8
Iran	1	1
Legal status		
Refugee	2	4
Asylum seeker	4	5
Other	1	
Time in Greece (range)		
0–1 years	1	
1–2 years	1	
2–3 years	2	
3–5 years	3	9
5 +	2	
Marital status		
Married	6	3
Single	3	6

^a^Age is given in five-year brackets to protect the confidentiality of the participants

We were assisted by three coordinators employed in two AOs offering their support as part of their regular work responsibilities without receiving any financial or other compensation for their involvement. These AOs were selected because of their operations in mainland Greece in both camp and urban settings, and their extensive networks with other AOs. The coordinators had higher education studies in migration or/and mental health. The coordinators had no personal relationships with the participants recruited for the project. The researchers informed the AO coordinators of the aims of the study. The coordinators facilitated participant recruitment by distributing information, and they ensured a private space with internet access for conducting the FGIs. They were not present during the FGIs. Interpreters assisted during the FGIs when needed. The interpreters signed a confidentiality agreement prior to the interviews. A short meeting between the interviewers and the interpreters followed each FGI to clarify potential misunderstandings or incidents.

The researchers emailed potential participants, informed them about the research, and invited them to participate. The participants were engaged in various ways as PRHs. At the time of the interviews, all were working, in paid or non-paid positions, for an AO in Greece. Their work involved various responsibilities, such as providing psychological interventions, coordinating practical activities and skill development courses for adults, engaging children in art courses, interpretation, and cultural mediation.

### Interview guide

We developed an interview guide (see supplementary file), informed by the salutogenic approach of our theoretical framework, which included the following main topics: *“Please tell us about your experience in helping others: Positive experiences you have had and what impact it had on you? Challenging experiences you have had and their impact on you? When facing difficulties*,* what has helped you so far? What helps you keep up being a helper in spite of adversity? Do you think about yourself in a different way now and how?”.* Additional topics addressed in the interview were informed by the first author’s experience working in the humanitarian field.

The interview guide was translated from English to Dari through a professional agency and then translated back to English by a native Dari speaker with a refugee background. At a consensus meeting with the first and second authors and the native speaker, we finalized the guide.

### Procedure

The data collection took place from the 1st of April 2021 to the 8th of June 2022, involving seven semi-structured, gender-divided focus group interviews (FGIs), each engaging two to four participants. Five FGIs were conducted online or in a hybrid format, meaning that some of the participants were in the same location while others joined digitally. COVID-19 pandemic restrictions led us to conduct most of the FGIs online, adapting to the current measures in Greece. Two FGIs took place face-to-face. We worked with interpreters in three FGIs where participants did not speak fluent English. The languages spoken in the interviews included Greek, Dari, and English. Six FGIs were conducted by the first author, and one FGI jointly with the second author.

The moderators encouraged open discussion. The average FGI duration was 73 min (minimum 65, maximum 91). FGIs were audio-recorded, transcribed verbatim by the research team, six by the first author and one by a research assistant, and anonymized. FGI digital recording files were stored on a secure server at the University of Bergen.

### Analysis

The data were analyzed in NVIVO 12 using template analysis [12], consistent with the design of our study and our epistemological position. According to template analysis, we defined concept-driven, a priori codes reflecting protective and risk factors for mental health in line with the salutogenic framework. All authors worked individually using these codes. During the course of three iterations, the authors discussed relevant theoretical frameworks in light of the adjusted codes. Specifically, the concepts of role overload and role conflict were found to fit the data well. A third category related to job stress was labeled role boundaries. This code refers to frustrations emerging from how their roles were defined by the AOs, as well as unclear expectations consistent with the concept of “role ambiguity”. The data were further considered to fit the theoretical model of the sense of coherence with the component’s three dimensions: meaningfulness, manageability, and comprehensibility. The final template (see Table 2) was applied to the full data set and found to effectively cover most of the data. The number of FGIs was determined after noticing recurring themes and reaching data saturation. The reporting process followed the consolidated criteria for reporting qualitative research [49].

Experience was valued in the process of our study. To this end, a reference group was established consisting of stakeholders involved in humanitarian services for refugees in Greece, including NGO representatives, policymakers, and members of refugee communities. The reference group provided input about the data collection and the interpretation of the results, contributing to the contextualization of the findings. Second, two female PRHs with Afghan refugee backgrounds provided feedback to a preliminary results meeting with the first author.

### Ethical considerations

As the Norwegian Regional Ethical Committee concluded that no health-related data were to be collected, it was exempted from review. The Norwegian Center for Research Data (Ref. number: 119451) approved the processing of personal data in accordance with data protection regulations. Efforts were made to minimize potential discomfort for the participants. As moderators, the first and second authors employed their skills as clinical psychologists to address any discomfort experienced by participants during and after the interview. Participants who expressed psychological distress were informed about available mental health resources. No information was shared with AOs without participants’ consent.

Before the interview, participants signed an informed consent form and further voiced their consent in the audio recording. In online or hybrid interviews, the AO collected and safely stored informed consent documents under lock and key until they were handed in person to the first author. In face-to-face interviews, the first author collected in person the participants’ signed informed consent forms in person. The AOs are not mentioned by name to protect the anonymity of the participants. Participants either read the consent forms themselves or had them read aloud by the interpreter. The first and second authors ensured the use of accessible language, with the assistance of an interpreter where necessary, to convey complex meanings to ensure that participants fully understood their rights and the nature of the research in which they participated.

## Results

The template analysis identified personal and work-related resources reflected in the SOC, as well as job demands for PRHs (see Table 2). We first exemplify how the three SOC components (meaningfulness, manageability, and comprehensibility) are reflected in themes addressed by the participants in the interviews (see Table 3). We then exemplify how job demands, reflected in informants’ experiences of role overload, role conflict, and role boundaries, were addressed (see Table 4).

**Table 2:** Final template of themes identified

1. Sense of Coherence	2. Job demands
1.1 Meaningfulness: Helping one of your own and yourself.	2.1 Role overload and emotional demands.
1.2 Manageability: Learning new skills.	2.2 Role conflict: adverse reactions from peers and responses.
1.3 Comprehensibility: Making sense and feeling safe	2.3 Role boundaries.

**Table 3:** Sense of coherence themes with illustrative quotes

Theme	Illustrative quote
**Meaningfulness: Helping one of your own and yourself.**	*“If you help one person*,* you get a lot of energy*,* and this energy doubles” [M1*,* MFG3*^a^ *].*
**Manageability: Learning new skills.**	*“We teach* [them] *some strategies we learned from the organization. There was so much to improve ourselves and to be a helpful person in society”* [M2, MFG1].
**Comprehensibility: Making sense and feeling safe.**	*“I feel a very clear feeling when I am working and helping other people. I know what I am doing. What I am giving to them. It makes me have a very clear view of the things” [F3*,* FFG1].*

^a^Gender and number of the participant, Code of the FGI.

**Table 4:** Job demands themes with illustrative quotes

Theme	Illustrative quote
**Role overload and emotional demands.**	*“When I go to a refugee’s house*,* I cry with the beneficiary*,* the refugee. Because when they tell their story*,* I cannot control myself” [M2*,* MFG3].*
**Role conflict: adverse reactions from peers and responses**.	*“When you go with the car somewhere to take something people look badly at us*,* saying*,* “where is she going with this car and this guy?”” [F2*,* FFG4].*
***Role boundaries***	*“Problems are not coming between 8 to 9. Problems are coming accidentally. They come at one o clock at night. Should you turn down people and say: “it’s not my time to work?” or “it’s not a good time for you to call me?”” [M1*,* MFG3].*

### Sense of coherence

#### Meaningfulness: helping one of your own and yourself

We understand meaningfulness here in perceiving demands as challenges worthy of engagement. One stated, *“I really*,* really*,* understand what they are feeling. The experience that if [you] haven’t had it [yourself]*,* you will never understand what is going on”* [M3, MFG1]. The common experiences with their beneficiaries thus increased their motivation. One stated, *“sometimes it’s out of my hands and I want to try. I want to try to support the beneficiaries. I am their refugee”* [M2, MFG3]. This process of providing support, in turn, seemed to increase the PRHs’ own sense of wellbeing. One stated, *“If you help one person*,* you get a lot of energy and this energy doubles” [M1*,* MFG3].*

Female participants specifically suggested that taking up an active role as a PRH gave them new life opportunities. One stated: *“If we always stay victim*,* we will not do anything*,* but we should accept our life and be responsible and do something. It’s going to be a big change for us and make a big difference”* [F2, FFG4]. Several female participants mentioned that helping others helped redefine themselves beyond the traditional roles of being a wife and a mother typically that were available to them, for example:

*I said I didn’t have freedom because*,* in Iran and Afghanistan*,* women have no value. The only things that they can do are stay at home and do the house chores. We didn’t have any freedom*,* just stay home and take care of the children (…). When I came to Europe*,* I could see that I have the power to do many things*,* I can learn a language*,* even I can be like my husband*,* the one that brings money to the house*,* brings income”* [F2, FFG2].

Male PRHs emphasized that assuming the role of a PRH improved their emotional wellbeing, instilled hope for the future, and provided opportunities for fulfilling personal goals, both within their families and in society. For example, one stated: *“When I began to help others*,* it improved me. And I became hopeful to have a solution*,* to have a job*,* to be responsible to help others”* [M2, MFG1]. Relating to being a good example, including being a good parent, one person mentioned how his PRH role also reflected on his role in the family: *“What I want from my life is for my children to learn from me what is the correct path”* [M1, MFG2]. Another participant, reflecting on his role in society, expressed, *“One drop from a big ocean. We are trying to be better and if all the NGOs and the other human beings see us*,* they can make an example out of us”* [M1, MFG3].

Thus, both male and female participants emphasized that being a PRH provided an opportunity to improve their situation, yet its impact exhibited clear gender differences.

#### Manageability: learning new skills

In this context, manageability refers to having or obtaining the necessary resources to deal with job demands. In our data, this aspect seemed closely related to learning new skills, which the PRHs, in turn, taught to others. One stated:

*“We help people. We teach people some strategies for them to find solutions for their problems. Stress*,* anxiety*,* and other things they have problems. So*,* we teach* [them] *some strategies we learned from the organization. There was so much to improve ourselves and to be a helpful person in society”* [M2, MFG1].

Another stated: *“It’s the best*,* the best experience in my life that I received from this organization to know how to handle the problems that we have daily” [M2*,* MFG1]*.

Supervision sessions in which PRHs discussed challenges with a mental health professional or a more experienced aid worker enhanced their feeling of being supported by the AO. One stated: *“We discuss what happened during the day*,* (…) and see what we can do better for the next day” [M1*,* MFG3].*

Several participants emphasized that they acquired practical skills through the PRH role. A male participant elaborated: *“An example is painting. I did not know how to paint. I started learning a bit. Then I helped those who knew less than me. At the same time*,* you learn yourself and you help others”* [M4, MFG2]. A female participant mentioned: *“I was talking to them*,* and I tried to speak English and improve my level of language. And I said: “If you need any help*,* I am here”* [F2, FFG4]. Another participant emphasized the benefits of learning about other cultures and developing more inclusive views on others: *“Sometimes we have incorrect views about another nationality. About another community. But when we are speaking together*,* when we are working together*,* we can get to know each other better”* [F3, FFG3].

#### Comprehensibility: making sense and feeling safe

Comprehensibility is related to the process of gaining a better understanding of their role and experiencing a more predictable and structured life. Ultimately, this seems to contribute to the PRH’s feeling of safety in the host country. Specifically, participants emphasized how their lives made more sense after assuming the role of PRH. One stated, “*I feel a very clear feeling when I am working and helping other people. I know what I am doing. What I am giving to them. It makes me have a very clear view of the things*” [F3, FFG1].

Living in volatile environments, PRHs acknowledge the crucial role that AOs play in ensuring their safety, especially during times of adversity. One participant explained: *“This organization really helped us a lot in [name of camp]. Because whenever there was trouble like a fire or a fight*,* they immediately communicated with us. The moment they knew something was wrong they would come with their car and pick us up telling us to stay away to be safe” [F1*,* FFG2].*

Understanding better how AOs work, further increased PRHs’ sense of safety. One stated:

*“Neither as a refugee nor as a local person will you feel insecurity when you are working with an organization at least for 6 months. Because you are going to receive a short-time contract*,* after your first-time contract. Possibly they are going to renew it” [M1*,* MFG1].*

A sense of safety was associated with increased engagement according to several participants. One stated, *“I have received a lot of offers from humanitarian organizations that ask for interpreters*,* but I say no. I am in my paradise. [Place of work] is my safest place*,* and I am not giving it up for the world” [M1*,* MFG3].*

### Job demands

#### Role overload and emotional demands

In this context, role overload is relevant to the challenge the PRHs reported when listening to beneficiaries share emotionally challenging life stories with them. Several expressed a sense of inadequacy in handling the impact these stories had on them.

One stated: *“Everyday it was more than a hundred people in line. Just imagine hearing these things thirty*,* forty times per day”* [F2, FFG1]. Listening to the experiences of the beneficiaries, many of which resonated with the helpers’ background, could increase psychological distress for both female and male helpers. One stated, *“The whole of their family was killed in Afghanistan. And their house was burned. And they did whatever they could. (…) I was unable to explain*,* it was reminding me of my own. A trigger warning for me”* [M1, MFG3]. Another described how they felt losing control as they empathized with the other refugees, *“When I go to a refugee’s house*,* I cry with the beneficiary*,* the refugee. Because when they tell their story*,* I cannot control myself”* [M2, MFG3].

#### Role conflict: adverse reactions from peers and responses

Role conflict in this context refers to the conflicting expectations from different sources, including disparities between work roles and social norms, including but not excluded to, socio-cultural gender roles that many are expected to live up to. This theme was most prevalent among the female PRHs. Several female PRHs, working in both camps and urban settings mentioned that they had been the target of negative, gendered reactions. One shared her experience from a camp: *“It was a lot of people*,* mostly single men. They were like*,* yeah*,* sometimes harassing*,* sometimes not”* [F2, FFG3]. Another, working in an urban setting discussed the reactions of males, “*It was really helpful for me*,* and at the same time it was really tough. Because in our community it’s difficult for the women to work with the men” [F2*,* FFG4].*

Certain activities, such as accompanying someone to various services, were mentioned as especially challenging for female PRHs. *“When you go with the car somewhere to take something people look badly at us*,* saying*,* “where is she going with this car and this guy?”” [F2*,* FFG4].* Additionally, some women reported uncomfortable interactions and harassment from male beneficiaries, *“They come close to me when I work there*,* and I hate it. I do not know how to tell them. We also have a class about self-defense. To be able to tell them “Step back”. I am trying” [F1*,* FFG4]*. The same PRH stated that her responses to harassment could potentially lead to retaliation: *“When I say something*,* they say something bad to me” [F1*,* FFG4]*. Another female PRH, referring to her work in a refugee camp, also mentioned harassment directed at the women living there, especially when the camp became overcrowded: *“Because it was a lot of people*,* mostly single men. They were like*,* yeah*,* sometimes harassing*,* sometimes not” [F2*,* FFG3].*

Female PRHs argued that they tried to cope with harassment in various ways, which, in turn, could further compromise their wellbeing. Some participants expressed that ignoring harassment was the best option for coping: “*But you know I didn’t give up because it was really important for me. I didn’t… I tried to just ignore them” [F2*,* FFG4].* A female participant commented that *“being like a man”* was a way to be accepted by men and do the job that you like to do. Discussing prior experiences in Iran as an Afghan woman, she elaborated: *“I tried to be like a man. I used to act like them. For example*,* when they wanted to call each other at the big factory they whistle. And I started doing like that*,* whistling and for me it was really helpful. It worked. And I was really strict. You have to be like that” [F2*,* FFG4]*.

Several women mentioned that they asked their peers to accompany them as a way of coping with negative reactions. Additionally, some changed their clothes into what could be perceived as more conservative from their peers: “*I cannot have someone coming with me every time. I come to this area almost every day. Mostly in [central place in a city center] I use to change my clothes” [F1*,* FFG4].* Another shared the emotional distress, saying. *“I hate this to change what I want to wear” [F2*,* FFG4]*. Later the former participant clarified: *“It’s these kind of things [sexual harassment incident] that I try to avoid and wear something more… Like a hijab” [F1*,* FFG4].*

#### Role boundaries

Role boundaries in this study related to the frustration experienced around the definition of the PRH role, and particularly regarding the expectations and demands from the AOs.

Several participants felt that their personal refugee experience was not adequately acknowledged by the AO. One stated: *“I didn’t have a nice experience working with them because they were really treating people badly*,* and they would make us feel bad about how we came*,* how we experienced different things”[F2*,* FFG4].* Another identified the fixed working hours as restricting their ability to provide appropriate help. One stated, *“Our organization says that after a certain time*,* I have to turn off my phone. But I cannot turn off my phone. Someone might call. Once*,* someone called me because they wanted to commit suicide. […] How can I control myself and turn off my phone?” [M2*,* MFG3]*.

Some helpers explained that they hoped to receive returns for their services through material rewards from the AO to help them cope with their adverse living conditions. However, they often faced strict limitations on the benefits they could receive in return. The lack of perceived reciprocity was indicated as a source of frustration, as many helpers viewed their role as critical to the functioning of the AO. One stated:

*“Also*,* when I was working with this organization in [name of place] sometimes*,* I was thinking about [getting] work*,* maybe improving my language and get something from this organization but honestly it was not like that. They didn’t give anything to the volunteers because they said: “Because you are a volunteer it’s not fair to give something to you”. But I used to work twelve hours. From 7 am to 7 pm” [F2*,* FFG4].*

Financial insecurity is tied to role boundaries since it reflects the often unclear relationship between paid and volunteer work. One stated, *“I was just volunteering. Now I am kind of*,* kind of paid but I don’t get proper money. I don’t have a job full-time or something because they [the NGO] cannot afford it” [F2*,* FFG4].* That is further explained by another helper, *“I worked with [name of organization] and they […] cannot give us a*,* like… broad contract but I am working with them*,* and I have a salary but it’s not […] it’s not salary*,* it’s not official. I am working as a volunteer with them. Just this. Honestly*,* it works for me like that” [F1*,* FFG4].* This sense of being content with payment often corresponded with covering basic needs that would otherwise be compromised. One stated, *“I am working here*,* and also*,* it’s a kind of volunteer job*,* but I can now see that here my basic needs are little. I don’t struggle with my basic needs” [M1*,* MFG3]*. It is further clarified by the same participant, *“I am getting a stipend. I am not getting*,* how do you say it*,* legally paid. It’s a black market for me. They give me my stipend so I can just survive. Pay my rent*,* have some for food” [M1*,* MFG3]*.

Unclear role boundaries also arose because of the AO’s policies on distribution procedures. Sometimes, the distribution procedures were a source of frustration, as they were perceived to prevent the helpers from meeting the needs of the beneficiaries. One person stated,

*“When you are working with NGOs*,* you are not allowed to help people. Not allowed to give any food*,* anything to them*,* to the person you know. Because of the NGO policies. Sometimes even you feel like there’s a child hungry*,* a family hungry and I want to do something*,* but you are not allowed to do it. You are not allowed to cross the boundaries” [F3*,* FFG1].*

Several participants expressed that their wellbeing was negatively affected by the constraints placed on the assistance they could offer to their beneficiaries, as dictated by the AO. One stated, “*Many times this is something you carry to your home. While I was eating food*,* they were in my mind. When my child was eating food*,* they were in my mind. That was one of the really very negative parts of working with the population because you are not allowed to do many things*” [F3, FFG1].

Another participant highlighted that appearing weak and in need was an informal requirement for refugees to receive help from AOs. As he argued:

*“Why do they have to make their stories sad? Because it’s the fault of the system. Consider that there are hundreds of NGOs in [name of place]. If you go to the same NGO and you say for example “I am a good person*,* a strong human being. My situation is not that difficult now*,* but I need this*,* and I need that can you give it to me?”. They will say to me “Shut up and just leave. You are a healthy human being and so strong*,* why should we help you? We are helping the people that are in a bad situation”. I am in a bad situation*,* but I am strong. I try to keep myself up and not down. It is the fault of the system” [M1*,* MFG3].*

The participant highlighted his disempowering experience when seeking help, as he was not considered “vulnerable enough” to receive aid. This underscores a broader issue within the system, where aid is often prioritized for those in the most visibly dire circumstances. Despite being in need, his resilience would lead to his dismissal by the AO. This experience is particularly significant since he values empowering beneficiaries. However, he often perceived that his values conflicted with the expectations of the AOs he had worked with. Finding an organization that aligned with his values was important for the participant, even though it meant accepting lower financial compensation, as was the case for him.

## Discussion

This is the first study to explore both positive and negative impacts of being a PRH on the mental health and wellbeing of Afghan refugees and asylum seekers. The themes identified through qualitative analysis encompassed the three components of SOC, as well as three distinct job demands, role overload, role conflict, and role boundaries. The results related to SOC appeared to contribute to job and life satisfaction, increased motivation and engagement, and hope for the future. In contrast, the job demands seemed to have the opposite effects. This is in line with a systematic review examining the relationships between SOC and job stress [22].

Our analysis revealed a complex and nuanced interplay between SOC, job demands, and their impact on wellbeing. For several women, being a PRH offered an opportunity to break free from what they perceived as restrictive gender roles and facilitated greater access to resources and a consequent sense of increased control over their lives. However, female PRHs also reported experiencing role conflicts between work expectations and traditional gender roles endorsed by several Afghan communities, which led to stress and frustration. We further elaborate on gender differences under each relevant theme. The differences in experiences between men and women PRHs represent one of the significant findings of this study.

### Positive effects and SOC: meaningfulness

The participants in our study reported an increased sense of meaningfulness through the PRH role. Meaningfulness was associated with the help PRHs provide and the active role PRHs hold in their respective communities. Aligning with our findings, a study among Afghan refugees living in Greek camps suggested that many experienced supporting and helping other refugees enabled them to break cycles of depression and inactivity [31].

The PRH role seemed to be particularly important for female PRHs. Women expressed that they perceived taking on an active role and helping their peers as liberating, contrasting it to a life where many were restricted to staying at home and fulfilling family obligations aligning with traditional Afghan values. The empowering effect on women holding a PRH role is consistent with other research among PRHs providing care to survivors of gender-based violence in refugee camps [26]. Men participating in this study emphasized meaningfulness in the importance of being able to fulfil their personal goals and become good examples to others, including their children, after taking up a role as PRH.

### Positive effects and SOC: manageability

We identified manageability as a central theme in the interviews. The PRH role was seen as a possibility to get access to resources useful to navigate post-migration challenges. PRHs emphasized that their role, through training, supervision, and service delivery, helped them effectively learn new skills. They specifically mentioned their ability to care for their own mental health by applying learned strategies, managing personal challenges, strengthening their helping skills, and increasing cultural awareness. Some acquired practical skills, such as learning a new language. Our findings align with a study in Switzerland, where PRHs reported improvement in their mental health through the strategies they learned through their role [45].

### Positive effects and SOC: comprehensibility

PRHs explained that psychological and physical safety in their work, were crucial for their mental health, particularly in a precarious work environment. Having a clear sense of purpose and defined work assignments as PRHs helped them establish structure in their lives.

Globally, there is a steady increase in attacks on aid workers [3], and PRHs working in refugee camps often report feeling unsafe [26]. Against this backdrop it is important to highlight that AOs could serve as a safe workplace by providing a structured life and ensuring physical and emotional protection for their PRHs.

### Negative effects and job demands: role overload

Being a PRH is linked to an increased emotional burden due to heavy caseloads and the distressing life experiences of beneficiaries, which often mirror their own. This could trigger adverse reactions while providing services. Similar role overload was reported among PRHs delivering a scalable psychological intervention in France [47]. In that study, PRHs found it challenging to maintain the necessary emotional distance and reported experiencing psychological distress during and after their visits to beneficiaries [47].

### Negative effects and job demands: role conflict

Female PRHs meet gender-specific challenges that are often inadequately addressed. Among international humanitarian aid workers, sexual harassment is more frequently reported by female aid workers compared to their male counterparts, negatively impacting their mental health and wellbeing [36]. Similarly, in our study, female PRHs often encountered harassment or other adverse reactions from their community. These findings are consistent with those of Brea Larios et al. [11], who reported that many Afghan refugee women in exile faced strong social control, further exacerbating their distress. Our results further align with Stempel et al. [46] who found that Afghan men adhering to conservative values experienced distress as they struggled to adapt to more egalitarian gender norms, and often actively restricted Afghan women from expanding their roles.

### Negative effects and job demands: role boundaries

Role boundaries in this study relate to frustration arising from an unclear PRH role and the imposed expectations and demands from the AO. For instance, many PRHs are asked to work without compensation or restrictions on working hours. PRHs are further challenged when asked to provide assistance without adequate support to address the pressing needs of the beneficiaries. Financial insecurity among PRHs is reflected in the lack of formalized employment, inconsistent low pay, and ambiguous expectations regarding the benefits of volunteering. These factors further contribute to blurred role boundaries and increased frustration among PRHs. Consistent with our findings, a recent review among peer support workers indicated that role boundary challenges negatively impact mental health [16]. We further elaborate on role boundaries and associated challenges.

First, the PRHs reported being treated differently than other aid workers, perceiving discrimination based on their refugee background in their workplace. Similar experiences of inequalities among aid workers were reported in an ethnographic study among aid workers in Kenya [43]. Specifically, Sackett [43] documents how international and national aid workers enjoyed more privileges than PRHs, which negatively impact the latter. Second, inadequate explanations of the operational procedures and expectations often conflicted with how PRHs perceive their role. Similar findings are reflected in Surkan et al. [47] where PRHs reported frustration when they experienced the boundaries set by the intervention as limiting their ability to help beneficiaries. Sackett [43] noted that PRHs often find themselves caught between the expectations of their community and those of the AO they are associated with, often reporting backlash from their peers when PRHs choose to adhere to the established limits of their work. Third, boundary challenges that affect PRH mental health are exacerbated by financial insecurity, lack of formalized employment, unclear expectations from volunteering and inconsistent low pay. International and national aid workers report negative impacts associated with low income [55]. The precarity that PRHs find themselves in, given their refugee background, is particularly important to highlight, as it may exacerbate the negative health effects of uncertainty in post-migration settings [33].

## Limitations

There are several limitations to our study. First, the sample of participants in the survey might not be representative of the PRHs who worked in the humanitarian field in Greece at the time. The study included a small sample size and only PRHs of Afghan origin, highlighting that caution should be taken when generalizing findings. Purposive sampling was employed to recruit participants. This method has been used in other studies with refugee populations, aiming to recruit participants with relevant experience in the research issue [1, 28].

Second, the language barrier should be acknowledged as a potential bias in our study. In the FGIs, we collaborated with interpreters translating from Dari to English or Greek. Due to different cultural backgrounds, non-verbal cues or idioms might have been misunderstood and other information might have been inadequately translated. In four FGIs, participants explicitly refrained from using interpreters and preferred to communicate in English. While this was accommodated, a lack of language proficiency may have limited the participants’ ability to express themselves. A potential reason for making such a request could have been concerns about whether the interpreters maintained confidentiality.

Third, the gender of the interviewer (male/non-binary) did not match the gender of the participants in two FGIs with female participants. The third FGI involving female participants was conducted by the same interviewer, along with a female interviewer (author GMS). In two of the three FGIs where an interpreter was necessary, the interpreter’s gender matched that of the participants. Due to pandemic restrictions, five FGIs were either online or hybrid (participants in the same place), and two took place face-to-face. To ensure data quality, we collaborated with the AOs to guarantee that the participants had privacy and reliable internet access.

Fourth, every FGI was facilitated by an AO providing a dedicated room for the interview. Although the PHR’s experiences did not always refer to the current AO they worked for at the time of the interview, there’s a potential social desirability bias in sharing mostly positive experiences.

Lastly, we acknowledge the limitations of using a theory-driven framework. Our data material was extensive and rich, encompassing a variety of insights and experiences beyond what we could include in this article. By choosing a theory-driven framework, we recognize that there are different aspects of the data which have not been included in this discussion.

## Conclusion

Engagement in peer support can be both rewarding and challenging for PRHs. Four recommendations can be made based on the findings of our study, informing both policies and practices in the humanitarian field. Firstly, we recommend that AOs facilitate empowering experiences for PRHs by regulating their workload and offering them support and training to enhance work engagement and prevent emotional exhaustion. Professional staff or experienced PRHs might support the PRHs, helping them navigate challenges and managing stressful reactions. Given the non-specialized background of PRHs, a competency-based framework in training and supervision aimed at developing concrete skills necessary for the PRH role has proven effective [39]. Additionally, psychosocial support from health professionals should be available to the PRHs, giving them the space to further process their own experiences while working with their beneficiaries. Secondly, it is important to consider gender differences by promoting awareness of the challenges faced by female PRHs and implementing concrete measures to address those challenges. It is important to address organizational culture and implement measures that promote comprehensive change [36]. For example, it is important to establish accessible mechanisms for whistleblowing, while further engaging with training and awareness-raising on gender-based violence in every level of the organizational hierarchy. Thirdly, AOs should consider adopting scalable psychosocial interventions which have shown to be effective for the refugee populations and culturally adapted to respond to their needs [13]. Such peer-led and community-based interventions, provide a concrete way of providing support and engaging with beneficiaries on the field promoting agency among engaged PRHs [2]. Lastly, job security and work engagement can be greatly facilitated through the implementation of formal contracts for both paid and volunteer PRH positions. These contracts should outline job roles and associated expectations. Additionally, it is essential to communicate this information effectively to ensure that PRHs fully understand the established processes and their rationale.

## Supplementary Information

Supplementary Material 1.

## Data Availability

Data are safely stored at a secure server (SAFE) at the University of Bergen as described at the Norwegian Center for Research Data approval (Ref. 119451). Due to ethical approval being contingent to the safe storage of data at our institution, we are prevented from having data available as supplementary material or transfer it to other repositories of data. Any requests for access should be sent to corresponding author.

## Abbreviations

PRH: Peer Refugee Helper
SOC: Sense of Coherence
AO: Aid/Humanitarian Organization
JD-R: Job Demands-Resources Model
