# Supplementary material for: “It was really helpful for me, and at the same time it was really tough”: a qualitative study among Afghan peer refugee helpers in Greece
Source: BMC Psychiatry. 2024 Nov 16;24:818. doi: 10.1186/s12888-024-06255-4 (PMC11568550; doi:10.1186/s12888-024-06255-4)
Supplement: Supplementary file 1 — Supplementary Material 1. [file 12888_2024_6255_MOESM1_ESM.docx]

**Interview guide for Peer Refugee Helper semi-structured focus group interviews.**

1. Introduction.
2. *Question:* What kind of experience in helping others have you had?
3. Positive experiences you had and what impact it had on you?
4. Challenging experiences you had and their impact on you?
5. *Question:* When facing difficulties, what has helped you so far?
6. *Question:* What helps you keep up being a helper in spite of adversity?
7. *Question:* Do you think about yourself in a different way now and how?
